# Winners and Losers in Palliative Care Service Delivery: Time for a Public Health Approach to Palliative and End of Life Care

**DOI:** 10.3390/healthcare9121615

**Published:** 2021-11-23

**Authors:** Samar M. Aoun, Robyn Richmond, Leanne Jiang, Bruce Rumbold

**Affiliations:** 1 Perron Institute for Neurological and Translational Science, Nedlands, WA 6009, Australia; robyn.richmond@perron.uwa.edu.au (R.R.); leanne.jiang@latrobe.edu.au (L.J.); 2 Public Health Palliative Care Unit, School of Psychology and Public Health, La Trobe University, Melbourne, VIC 3086, Australia; b.rumbold@latrobe.edu.au

**Keywords:** palliative care, end of life care, consumer perspectives, equity, public health approach, compassionate communities, caregiving, bereavement

## Abstract

Background: Consumer experience of palliative care has been inconsistently and selectively investigated. Methods: People in Western Australia who had experienced a life limiting illness in the past five years were recruited via social media and care organisations (2020) and invited to complete a cross sectional consumer survey on their experiences of the care they received. Results: 353 bereaved carers, current carers and patients responded. The winners, those who received the best quality end-of-life care, were those who were aware of palliative care as an end-of-life care (EOLC) option, qualified for admission to and were able to access a specialist palliative care program, and with mainly a cancer diagnosis. The losers, those who received end-of-life care that was adequate rather than best practice, were those who were unaware of palliative care as an EOLC option or did not qualify for or were unable to access specialist palliative care and had mainly a non-cancer diagnosis. Both groups were well supported throughout their illness by family and a wider social network. However, their family carers were not adequately supported by health services during caregiving and bereavement. Conclusions: A public health approach to palliative and end of life care is proposed to integrate tertiary, primary, and community services through active consumer engagement in the design and delivery of care. Therefore, suggested strategies may also have relevance in many other international settings.

## 1. Introduction

A recent literature review on consumers’ needs and preferences [1] found that consumer experience of palliative care has been inconsistently and selectively investigated. The review highlighted that, in addition to competent treatment, information about the illness experience and strategies for managing that experience in everyday life are important to patients and their family carers. It also indicated that end-of-life needs usually include needs that have already arisen earlier in patients’ illness journeys. For some patients, transfer to an end-of-life care setting to meet fresh needs that arise can have the unintended consequence that important continuing needs are no longer adequately met [1]. It has been suggested that such inconsistencies and disruptions in care could be addressed by implementing a patient journey perspective that requires services to be integrated across the illness course [2,3].

Tools assessing how patients prefer to engage in healthcare are of variable quality [4] and need further consumer involvement in their development. Consumer experience studies that indicate the process of engagement and negotiation are in themselves helpful in building relationships and reinforcing support [5,6,7].

Most palliative care programs collect some form of internal consumer data, but few publish their findings. Consumer satisfaction scores are often collected by palliative care peak bodies, but this activity tends to be more a marketing measure than a focused enquiry into quality. To date, the only program that has consistently collected consumer data in granular detail across a health system is the Views Of Informal Caregivers Evaluation of Services (VOICES), commissioned by the UK Department of Health [8], and badged as the National Survey of Bereaved People once managed by the Office for National Statistics. This UK survey investigated the quality of care delivered in the last three months of life for adults who died in England, using a sample of approximately 30% of all deaths over a four-month period selected from the death registration database. One of the strengths of the national survey has been its contribution to quality at the local level, although these local projects seldom lead to peer-reviewed publications.

The closest equivalent to VOICES in Australia is the FAMCARE-2 tool (Family Satisfaction with Palliative Care), which measures satisfaction across four domains, management of physical symptoms and comfort, provision of information, family support, and patient psychological care. The validation study [9] indicated lower levels of satisfaction in response to the subscales ‘provision of information’ and ‘family support’, consistent with VOICES findings. FAMCARE-2 has been adapted to MND Care to evaluate consumer satisfaction [10]. FAMCARE-2 is administered periodically in selected services by Australia’s Palliative Care Outcomes Collaboration (PCOC). A survey of 1592 caregivers across 49 palliative care services in 2016 [11] found generally high levels of satisfaction and positive experiences of care. Scores were higher for in-patient care on three of the four domains, provision of information being the exception. Dissatisfaction with information provision was higher for older carers, while home carers reported that information to support them with practical caring tasks was inadequate. Similar results were found with the Australian EOL and Bereavement Study [12].

The literature review on consumer needs and preferences also found that, while contributions from consumer engagement were virtually absent from policy documents and service guidelines documents a decade ago, they have in recent years been consistently included [1]. These contributions are, however, uneven. Recent documents acknowledge the importance of community involvement or engagement although few expand on how this engagement might take place. Despite support for community engagement and involvement of informal carers, most current models still fall short because their integration is limited to formal health services, with consumers consulted as clients rather than partners in the co-design of services. The review proposed public health approaches to palliative care as a framework for addressing these shortfalls [1]. The aim of this article is to report on what matters most to consumers (patients and informal carers) in the delivery of palliative care in Western Australia.

## 2. Objectives

This study was commissioned by the Western Australian Department of Health’s End of Life Care (EOLC) Program as an independent review (i) to gain a consumer perspective on palliative care, (ii) to identify key challenges/gaps in the provision of EOLC, and (ii) to determine how service delivery can adapt and improve to meet community needs and expectations. The independent review took place from May to November 2020 and comprised three phases with the same three above-mentioned objectives: a literature review, a cross sectional consumer survey, and consultation forums with service providers. This article reports on the findings of the consumer survey where the winners and losers in palliative care service delivery are highlighted regardless of the care setting. A forthcoming article will focus on experiences specific to care settings such as home, hospice, hospital, and nursing home.

## 3. Methodology

A cross sectional consumer survey was designed to respond to the six priorities of the Western Australia (WA) End of Life Strategy [13] for developing and improving palliative care services across WA:

Priority 1. Care is accessible to everyone, everywhere
Priority 2. Care is person-centred
Priority 3. Care is coordinated
Priority 4. Families and carers are supported
Priority 5. All staff are prepared to care
Priority 6. The community is aware and able to care

The survey contained sets of questions grouped under the six priorities and related to the following: patient and carer demographics; experience with and quality of care in separate settings (home, hospice, hospital, and nursing home); formal and informal support before death, at the time of death, and after death. A number of questions in the sections on experiences in the four settings were adapted from the UK VOICES annual survey [8].

The survey was available in six formats for six different target consumer groups who had experienced a life limiting illness in the last five years: Consumers (patients and family carers) who are current or past users and non-users of palliative care services, were invited to report on what is working and not working well (or what has or has not worked well or could have worked better) for them in their end-of-life experience.

The survey was made primarily available as an online survey using Redcap. Hard copy surveys were also provided on request. Complementing the survey tool were additional documents for respondents to clarify several aspects of the survey: definitions of several terms used in the field; a list of palliative care service providers in WA categorised by setting; information on palliative care information and services; information on grief and bereavement, including how to contact services should the respondent become distressed completing the survey; and a participant information sheet. The survey was promoted extensively, but over a short six-week period, via service providers and relevant social media pages.

Ethics approval was granted by La Trobe University Research Ethics Committee (HEC20232). As this was an anonymous online survey, returning the completed survey was considered as implied consent. The information sheet that accompanied the survey emphasised that participation was entirely voluntary.

**Figure 1:**
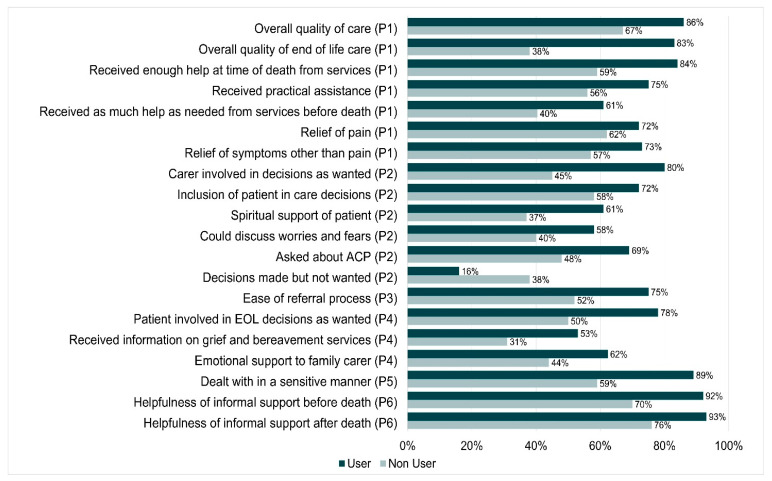
Differences in care between users and non-users of palliative care services (with EOL strategy priority number).

**Figure 2:**
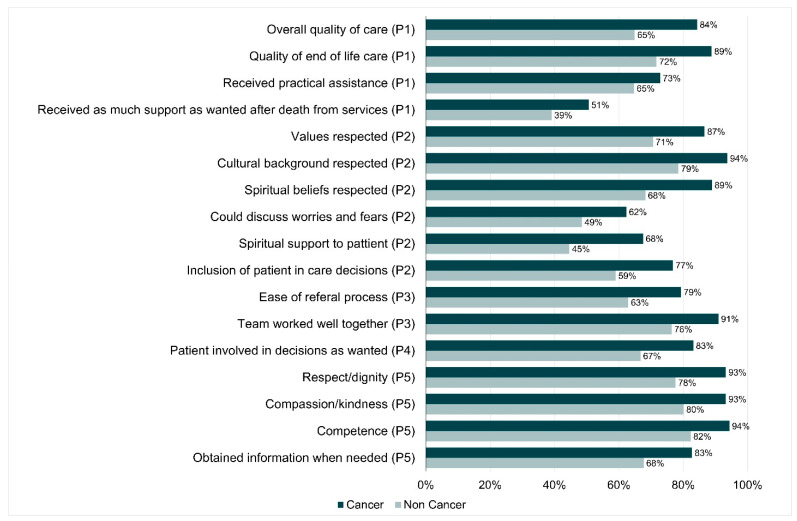
Differences in care between cancer and non-cancer conditions (with EOL strategy priority number).

Descriptive analyses were undertaken. Statistical tests for significant differences in quality indicators between users/non-users and cancer/non-cancer groups could not be performed as participants contributed multiple responses for settings of care and therefore violated the statistical assumption of independence. However, we have indicated in Figure 1 and Figure 2 where differences are large enough to be worth considering.

## 4. Results

### 4.1. Profile of Survey Respondents

In total, 430 surveys were received, with only three as paper copies. Following data cleaning the final number suitable for analyses was 353: 71% of total respondents were bereaved carers; 68% of total respondents had used a palliative care service; The most reported setting for care was home (43%) followed by hospital (26%), hospice (23%), and nursing home (8%). The majority of respondents used one care setting. There were no significant differences in the demographic characteristics of the users and non-user groups. Focusing on the largest respondent group, the bereaved carers, for both users and non-users, about 90% were female, average age was 55 years, over 50% had university level education, and over 60% were working. There were some slight differences in marital status (34% widowed users vs. 22% widowed non-users), rural/regional residence (28% users vs. 18% non-users), and relationship to patient (spouse/partner 34% users vs. 24% non-users). However almost twice the number of non-users had non-cancer diagnoses, mainly MND, dementia, and lung disease (27% users vs. 56% non-users). Table 1 presents the distribution of survey respondents across the groups. Table 2 presents selected demographic characteristics of users and non-users of palliative care services.

### 4.2. Non-Users’ Reasons for Not Receiving Palliative Care

Fifty eight out of 114 non-users gave reasons as to why they did use palliative care services with 72 multiple responses. The most common responses were: Unaware of what was available (17%); Satisfied with the care we received from general health and community services (12%); Tried but could not access them (11%); No-one initiated a referral to these services (11%); We were told it was not close enough to dying/end of life stage (10%); We were told it was too soon in the illness trajectory (8%); Unaware how it could help (8%).

### 4.3. Differences in Quality Indicators within the Six Priorities

Table 3 summarizes what worked well and not so well for consumers. Aspects of care services were delivering very well (at 90% satisfaction) were the competence of staff, working well together within a care setting and treating consumers with respect/dignity and compassion/kindness. Taking into account culture, values, and beliefs, access to care, relief of pain, being involved in decision making at end of life came next (at 80% satisfaction), followed by relief of symptoms, practical assistance, patient included in care decisions (70%). However, consumers reported more needs not being adequately met in questions relating to priority four, about families and carers not being supported before and after death of their relative. Most quality indicators for non-users of palliative care services were lower than those of users and those for non-cancer conditions rated lower than those for cancer, as described in detail below.

### 4.4. Differences in Care between Users and Non-Users of Palliative Care Services

The most pronounced differences were in the following quality indicators where the non-users fared worse (Figure 1): quality of end of life care (83% users vs. 38% non-users); receiving enough help at the time of death (84% users vs. 59% non-users); receiving practical assistance (75% users vs. 56% non-users); relief of pain (77% users vs. 62% non-users) and other symptoms; carer involved in decisions as wanted (80% users vs. 45% non-users); inclusion of patient in care decisions (72% vs. 58%); spiritual support of patient (61% vs. 37%); could discuss worries/fears (58% vs. 40%), being asked if they had EOL wishes documentation (69% vs. 48%); twice as many non-users had decisions made that were not wanted (16% users vs. 38% non-users); receiving as much help as needed before the death (61% vs. 40%); However, the support received after death was equivalent in both groups (47% vs. 45%). The referral process to services was not as easy for the non-users (75% users vs. 52% non-users).

Only 50% of non-users reported that the patient was involved in decisions at EOL compared to 78% of users; 31% of non-users received information on grief and bereavement services compared to 53% of users; 44% of non-users reported family carer receiving emotional support compared to 62% of users; being dealt with in a sensitive manner (89% users vs. 59% non-users). Both groups received the same extent of informal support. The non-users however reported lower ratings for the helpfulness of informal support before death (70% vs. 92%) and after death (76% vs. 93%). These differences could be accounted for by the way palliative care services tend to be intentional about encouraging caregivers to look and ask for support, including from not-for-profit organisations, in a way that other services may not [12].

### 4.5. Differences in Care between Cancer and Non-Cancer Conditions

Across most indicators, quality indicators for non-cancer rated lower than cancer (Figure 2), especially overall quality of care (65% vs. 84%), quality of EOLC (72% vs. 89%), respecting values (71% vs. 87%), culture (79% vs. 94%), spiritual beliefs (68% vs. 89%), could discuss worries/fears (49% vs. 62%), spiritual support for patient (45% vs. 68%), patient involved in decisions as wanted (67% vs. 83%), ease of referral process (63% vs. 79%) and staff worked better together (76% vs. 91%), staff competence (82% vs. 94%), being treated with respect/dignity (78% vs. 93%).

### 4.6. What Could Have Worked Better for Consumers?

Suggestions for improvement from respondents included the following: being provided with earlier access to palliative care, assistance completing relevant documentation on advance care planning and having this followed by staff, improving consumer knowledge, improving referral processes, better delivery of pain medication, improving staff training and staffing levels, better after-hours access and support to stay at home, increasing the level of family carer support and consumer involvement in decision making and care choices, improving delivery of palliative care for non-malignant diseases and more specifically for neurodegenerative conditions such as MND and dementia, and better access to palliative care in rural and remote areas.

## 5. Discussion

This survey was a first of its kind in Australia in terms of its depth and breadth of community engagement. Of the six priorities, quality indicators for Priority Four (families and carers are supported) lag behind the others. Families are not well supported before and after bereavement. Of user respondents, 40% reported that emotional and spiritual support to their patient was not adequate. This is considerably lower than other indicators such as symptom management. Emotional support to family carers and ability to discuss worries and fears were not adequate for 40% of respondents, as also reported by Wang [14]. About 40% of bereaved respondents felt that they did not receive as much support as they wanted from palliative care services during the illness and 50% did not after the patient’s death. Support after death was generally not adequate [12,15]. Although these aspects (emotional and spiritual support for patient and family before death, and bereavement support) are promoted as part of the holistic approach of palliative care, there seems to be in practice a disconnect between what the sector portrays and encourages the community to expect and what is actually delivered by services. This may be in part due to time and resource constraints on service capacity, and in part because some of these end-of-life needs are by their very nature unable to be met by professional support [16].

One strategy to address this deficit in support is to develop better referral pathways to not-for-profit support organisations who can, with better resourcing, dedicate the time to have these conversations and provide this support [17]. However, alongside this is the need to upskill the community networks that support people throughout the illness journey. It is evident that family carers assumed the vital role of ‘connecting the dots’ in liaising with several treating teams and maintaining communication around coordination of care. In the absence of formal case managers, family carers must be supported to develop their capacity in this role if care is to be as effective as possible. Family carer support needs to be at two levels: to help family carers care for the ill person but also to help care for themselves [18,19,20]. This can be achieved by instigating a system that assesses and addresses carer support needs, collects regular consumer feedback, and co-designs service improvements.

By way of contrast, responses to Priority Six (the community is aware and able to care) gave high ratings to the care provided by informal networks. Over 90% of respondents (both users and non-users) relied on the community (family/friends/neighbors/community organizations) to support them before and after bereavement and reported that this informal support was helpful in attending to practical, social, emotional, and spiritual support needs. Other studies support this finding [19,20,21]. The primacy of support from social networks is at the heart of public health approaches to palliative and end of life care which have the potential to enhance integration of services and provide a comprehensive approach that engages the assets of local communities [1,22]. The disease-specific not-for-profit support organisations have an important role to play in this space and their role too should be bolstered [17].

A particular innovation in this project was to seek responses from non-users as well as users of palliative care in order to explore sources of help received by non-users and how these compared in quality to the help users received from palliative care services. In general, the quality indicators reported by non-users were lower than those of the users. However, it should be noted that non-cancer conditions made up nearly 60% of non-users and their quality indicators were consistent with those with non-cancer conditions who used palliative care services. This would seem to reflect that palliative care services tend, as a result of their evolution, to be expert in cancer care but may lack expertise in other life-limiting conditions [23]. Another recent retrospective study of cancer and non-cancer patients in a Canadian tertiary palliative care setting did not find any major distinction between the needs of the two groups [24], but they did note that the non-cancer patients, a small proportion of the sample, were admitted precisely because their physical needs matched the expertise of the service. Of course, patients with a cancer history have on average significantly greater engagement with the health system, usually through hospitalisation, than non-cancer patients [25], and this may also shape expectations of care.

It is encouraging to know that palliative care services make the experience of patients and families considerably better in most aspects of care and also that the palliative approach to care provided by general services can be effective [26]. However, the issue remains that palliative care services select patients on the basis of the services’ capacity to respond to physical symptoms most commonly found in advanced cancer patients [27], not the broader spectrum of end-of-life need found across the general population [14,28].

Given that equity in end-of-life care provision is a goal of government policy [1], a key finding of the survey is the lower standard of care for non-cancer conditions across all six priorities. Because the survey relied on social connections to gather data, it did not receive responses relating to people who were socially disconnected in their dying, where their quality measures on family and community support measures would be expected to be even lower than those measured here. Inequity arising from disease type should be addressed within the health system. Inequity arising from inadequate social support must be addressed by local communities. Both aspects are taken into account by a public health approach.

Non-cancer conditions would benefit from a more inclusive palliative approach based on partnerships between specialist and generalist services to ensure that expert end-of-life care is added to, but does not replace, expert care for the illness with which the person is dying [26]. Non-cancer conditions should be given specific attention in new models of integrated end-of-life care. Current service provision should be bolstered by systematic and consistent education and training for general health professionals to ease the pressure on specialist palliative care services, and to broaden the non-cancer expertise available to these services.

Models of integrated care need also to be grounded in community upskilling/knowledge, supported consistently by generalist palliative care, with specialist palliative care providing ‘episodic’ care as particular needs arise during the illness journey [29]. This includes mobilising community options/better primary care engagement and potentially episodic care to broaden the reach of palliative care, case coordination, and improved communication/clinical handover. The role of not-for-profit organisations is vital in this space.

A public health approach to palliative and end of life care is able to achieve an integration of tertiary, primary, and community services through active consumer engagement in designing and delivering care to provide a comprehensive approach that engages the assets of local communities. This approach constructs a framework in which partnerships can be developed with patient communities with distinctive end of life needs, such as those with non-cancer conditions, thus providing a more inclusive approach to EOLC [30]. To implement this approach, we need to hear directly from the consumers about their experiences of unmet needs and how these could be met with better partnerships between the health services and the community, with the consumer involved in the co-design.

**Figure 3:**
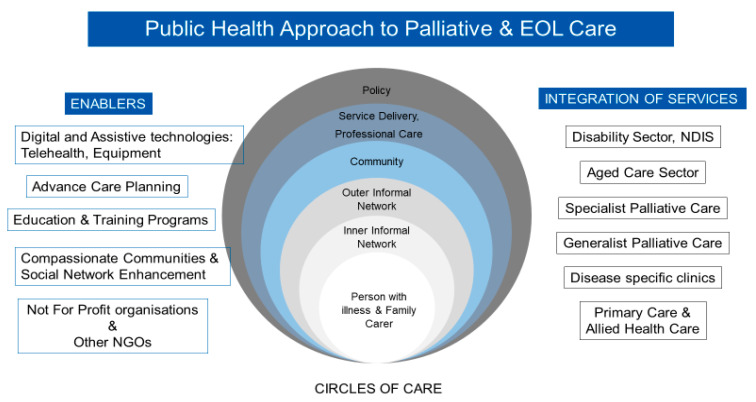
Public Health approach to palliative and EOL care.

This approach is illustrated in Figure 3 and recognises the ‘patient and social network’ (Circles of Care) [31]. The ‘inner’ and ‘outer’ circles of care, and neighbourhood supports are the main foundation of resilient networks caring for people at home. Together they form a Compassionate Community. However, these systems must also ensure that professional care, service delivery and policy enhance the care provided by the person’s social network. The model can be used as a practical guide about how care can be provided in communities and how different formal and informal services coordinate with each other and the communities they serve. The model looks at integration of the disability, health, and aged care sectors (tertiary, primary, and community services, specialist palliative care, generalist palliative care, disease specific clinics, and primary and allied health care services). The enablers for this integration include digital and assistive technologies, telehealth, advance care planning, education and training for health professionals and the community, not for profit organizations, and a compassionate community approach to care which enhances social networks.

### Strengths and Limitations

The sample that responded to the survey is not representative of all those who received specialist palliative care services in WA. A random or stratified sample would have been necessary to be truly representative but not at all achievable, especially given the methodological, ethical, and logistical challenges of conducting palliative care research [6,32], and the limited time available to produce findings from the three phases of this statewide review. Nevertheless, the findings where unmet needs exist are in line with those reported in the national and international literature [14,33]. The respondents to this survey have quite distinctive characteristics: 51% had a university education, 60% had one or more documents on EOL wishes, the majority with non-cancer conditions had dementia, MND or lung disease. That 60% of survey respondents who used palliative care services had a document on EOL wishes is encouraging as the rate of uptake in the general population is only 10% in WA. Perhaps there is a selection bias here in that those with an EOL wishes document may be more inclined to give their opinion on their experiences. Additionally, there is evidence that people using palliative care services are twice as likely to have an EOL wishes document completed [12]. Two-thirds of rural respondents were from a regional area close to the capital which consequently is better resourced than others, which is why rural–urban comparisons have not been included in this article. Nevertheless, there was good representation of 30% of rural consumers and those with non-cancer conditions, both of which tend to be under-represented in other studies. The respondents who self-selected to participate seemed to be well informed, had a keen interest to improve experiences for other people in their situation and were constructive with their suggestions for improvement.

There were five Aboriginal and Torres Strait Islander respondents, eight from culturally and linguistically diverse backgrounds and five from the paediatric population (<18 years). Given the low number of respondents in these three groups, separate analyses for them were not possible. While these population groups have been the subject of a more recent tailored national review into their needs [34], further efforts are needed to document and incorporate consumer feedback from these groups in prospective frameworks on end-of-life care.

## 6. Conclusions

This consumer survey has provided a detailed exploration of experiences during the caregiving journey through to bereavement, identifying strategies that worked well and strategies that could have worked better. The survey also provided useful feedback to services as to where they are meeting the six priorities of the Strategy and where there are still unmet needs as experienced by their consumers. It also provided some insight into those who die without accessing their services.

It is admittedly stretching the point to characterise respondents to the survey as winners or losers, but clearly differences in quality of end-of-life care emerged. The winners—those who received the best quality end-of-life care—were those who were aware of palliative care as an EOLC option; qualified for admission to, and were able to access, a specialist palliative care program; and were supported throughout their illness by family and a wider social network. They were for the most part people with a cancer diagnosis. The losers—those who received care at end-of-life that was adequate rather than best practice—were those who were unaware of palliative care as an EOLC option or did not qualify for or were unable to access specialist palliative care, and for the most part had a non-cancer diagnosis. They too were supported by family who responded to the survey. In the losers’ category are also family carers not being supported during caregiving and during bereavement.

A public health approach to palliative and end of life care is proposed to integrate tertiary, primary, and community services through active consumer engagement in the design and delivery of care. A public health approach to palliative and end of life care in the broadest sense encompasses a primary care approach involving generalist healthcare workers in providing initial assessment, support, intervention, and ongoing support; a tertiary care approach involving specialist healthcare providers and inpatient facilities such as hospitals, clinics, or hospices; and a population health approach involving education and community development. The latter is the least-developed aspect of palliative care service development. Hence the distinctive focus of a public health approach to end-of-life care today is that it views the community as an equal partner in the long and complex task of providing quality healthcare at the end of life.

This WA initiative of surveying consumer responses to EOLC, not simply palliative care, is an important step forward both in understanding quality of care at the end of life, and some sources of inequity in accessing end-of-life care. While the focus of attention in this article has been consumer experience in the state of Western Australia, the correlation of the findings with the international literature [1] suggests that strategies and solutions put forward for Western Australia may also have relevance in many other settings around the world. Further research including more socially diverse samples is required to identify the scope of need to be addressed by community care.

## Figures and Tables

**Table 1:** Distribution of survey respondents.

Number of Respondents	Used Palliative Care (Users)	Did Not Use Palliative Care (Non-Users)	Total
Bereaved Carer	204	45	249 (71%)
Current Carer	27	45	72 (20%)
Patient	8	24	32 (9%)
TOTAL	239 (68%)	114 (32%)	353 (100%)

**Table 2:** Demographic characteristics of survey respondents.

	Users						Non-Users					
	Bereaved		Current		Patient		Bereaved		Current		Patient	
	Carer		Carer				Carer		Carer			
	(*n* = 204)		(*n* = 28)		(*n* = 8)		(*n* = 45)		(*n* = 45)		(*n* = 24)	
	*n*	%	*n*	%	*n*	%	*n*	%	*n*	%	*n*	%
Gender												
Male	21	10.3	6	21.4	2	25	4	8.9	6	13.3	9	37.5
Female	181	88.7	22	78.6	6	75	41	91.1	39	86.7	15	62.5
Missing	2	1	0	0	0	0	0	-	0	-	0	-
Age, year												
Mean (SD)	54.6 (13.2)		46.5 (12.3)		66.7(9.3)		57.4 (12.2)		56.8 (11.3)		65.7(7.5)	
Median (Range)	55.0 (26–89)		46 (20–69)		69 (47–75)		57 (30–83)		58 (33–73)		66 (53–81)	
Marital Status												
Married/de facto	91	44.6	19	67.9	5	62.5	26	57.8	36	80	13	54.2
Widowed	70	34.3	1	3.6	0		10	22.2	2	4.4	3	12.5
Other	38	18.6	8	28.6	3	37.5	8	17.8	6	13.3	8	33.3
Missing	5	2.5	0	-	0	-	1	2.2	1	2.2	0	-
Education Level												
University	102	50.5	14	50	2	25	24	53.3	26	57.8	14	58.3
Below University	98	49	13	46.4	6	75	21	46.7	17	37.8	10	41.7
Missing	3	14.8	1	3.6	0	-	0	-	2	4.4	0	-
Employment Status												
Working	128	62.7	20	71.4	0	-	29	64.4	21	46.7	4	16.7
Not Working	24	11.8	6	21.4	2	25	5	11.1	11	24.4	3	12.5
Retired	49	24	1	3.6	6	75	11	24.4	11	24.4	17	70.8
Missing	3	1.5	1	3.3	0	-	0	-	2	4.4	0	-
Residential Postcode												
Metropolitan	140	68.6	18	64.3	4	50	34	75.6	35	77.8	17	70.8
Regional/Rural	56	27.5	9	32.1	3	37.5	8	17.8	10	22.2	6	25
Interstate	1	0.5	0	0	0	0	1	2.2	0	0	0	0
Missing	7	3.4	1	3.6	1	12.5	2	4.4	0	0	1	4.2
Relationship to Patient												
Spouse/Partner	70	34.3	5	17.9	-		11	24.4	24	53.3	-	
Daughter/Son	79	38.7	11	39.3	-		21	46.7	16	35.6	-	
Other	48	22.5	11	39.3	-		13	28.9	5	11.1	-	
Missing	7	3.4	1	3.6	-		0	-	0	-	-	
Disease												
Cancer	118	57.8	14	50.0	5	62.5	17	37.8	14	31.1	20	83.3
Non–Cancer	55	27	12	42.9	3	37.5	25	55.6	30	66.6	4	16.7
Motor Neurone Dis.	19	-	3	-	1	-	5	-	2	-		-
Dementia	14	-	4	-	0	-	7	-	18	-	0	-
Other neurological	3	-	1	-	1	-	8	-	8	-	2	-
Lung/Heart/Kidney	24	-	6	-	1	-	7	-	7	-	2	-
Missing/Unknown	31	15.2	2	7.1	0	0	3	6.7	1	2.3	0	0

**Table 3:** Differences in quality indicators within the six priorities.

What Is Working Well	What Is Not Working So Well
Priority one: Care is accessible to everyone, everywhere	
78% rated quality of care excellent to good	60% reported receiving as much support as wanted before death.
84% could access care as soon as they needed.	50% felt they received enough help after their relative’s death
76% rated relief of pain excellent to good	All indicators lower for non-cancer conditions
70% for relief of symptoms other than pain and practical assistance.	All indicators lower for non-users of palliative care
80% rated quality of EOL care excellent/good and reported they received enough help at time of death (definitely/ to some extent).	
Priority Two: Care is person-centred	
83% rated values respected always/most of the time	58% felt they could discuss worries/fears as much as they wanted
87% rated cultural background respected always/most of the time	61% rated spiritual support as excellent/good
82% rated spiritual beliefs respected always/most of the time	64% rated emotional support as excellent/good
69% reported that the services checked if they have EOL wishes documents	All indicators lower for non-cancer conditions
78% felt their wishes were taken into account	All indicators lower for non-users of palliative care
72% of patients felt included in care decisions (excellent/good)	
80% of carers reported being involved in decision making at EOL as much as they wanted	
Priority Three: Care is coordinated	
75% found the referral process easy/very easy	60% reported that services worked well with GP and external services
87% thought staff worked well within each setting (definitely/to some extent)	10% of ED admissions were planned or coordinated
74% rated out of hours services as excellent/good	All indicators lower for non-cancer conditions
Priority Four: Families and carers are supported	
78% reported patients were involved in decisions about their EOL care as much as they wanted	62% rated emotional support to family carer as excellent/good
	60% were provided information on their relative’s condition
	47% of carers reported being able to talk about experience of illness and death to services
	53% of carers were offered information on grief by palliative care services
	42% of carers were contacted by palliative care services 3–6 weeks after death and only 16% six months after death of their relative
	All indicators lower for non-cancer conditions
	All indicators lower for non-users of palliative care
Priority Five: All staff are prepared to care	
88% thought they were treated with respect/dignity always/most of the time	All indicators lower for non-cancer conditions
89% thought they were treated with compassion/ kindness always/most of the time	
90% rated staff as very competent/competent	
78% said they could obtain information when needed always/most of the time	
86% of carers reported being dealt with in a sensitive manner at death/end of life	
Priority Six: The community is aware and able to care	
96% reported they received informal support before death and 92% found this informal support very/quite helpful	Lower rates of helpfulness before and after death for non-users
94% reported they received informal support after death and 87% found this informal support very/quite helpful	

## Data Availability

Ethical approval precludes the data being used for another purpose or being provided to researchers who have not signed the appropriate confidentiality agreement. Specifically, the ethical approval specifies that all results are in aggregate form to maintain confidentiality and privacy and precludes individual level data being made publicly available. All aggregate data for this study are freely available and included in the paper. Interested and qualified researchers may send requests for additional data to Samar Aoun at s.aoun@latrobe.edu.au.
